# International Periodical Congress of Gynæcology and Obstetrics

**Published:** 1892-06

**Authors:** F. Henrotin

**Affiliations:** 353 LaSalle Ave., Chicago, Ill.


### INTERNATIONAL PERIODICAL CONGRESS OF GYNÆCOLOGY AND OBSTETRICS.

FIRST SESSION.—Brussels, Belgium, Sept. 14 to 19, 1892.

The following named distinguished gentlemen have been delegated to represent the British Gynæcological Society at the International Congress of Gynæcology and Obstetrics, next September: Robert Barnes, Granville Bantock, A. S. Simpson, Lawson Tait.

Great preparations are being made to entertain visiting physicians. His Majesty, King Leopold, will assist at the opening of the congress. There will be a grand reception by the Belgian Gynæcological Society; gala performance at the Grand Opera; also a banquet by the British Gyn. Society; garden party in the garden of the royal family, etc.

For all information relating to the congress, address,

Dr. F. HENROTIN,
353 LaSalle Ave., Chicago, Ill.

